# Turkish pediatric atypical hemolytic uremic syndrome registry: initial analysis of 146 patients

**DOI:** 10.1186/s12882-016-0420-6

**Published:** 2017-01-05

**Authors:** Nesrin Besbas, Bora Gulhan, Oguz Soylemezoglu, Z. Birsin Ozcakar, Emine Korkmaz, Mutlu Hayran, Fatih Ozaltin

**Affiliations:** 1 Department of Pediatric Nephrology, Nephrogenetics Laboratory, Hacettepe University Faculty of Medicine, Sihhiye, 06100 Ankara Turkey; 2 Department of Pediatric Nephrology, Gazi University Faculty of Medicine, Ankara, Turkey; 3 Department of Pediatric Nephrology, Ankara University Faculty of Medicine, Ankara, Turkey; 4 Department of Preventive Oncology, Hacettepe University, Ankara, Turkey

**Keywords:** Atypical hemolytic uremic syndrome, Turkish registry, Treatment, Outcome, Prognosis

## Abstract

**Background:**

Atypical hemolytic uremic syndrome (aHUS) is a devastating disease with significant morbidity and mortality. Its genetic heterogeneity impacts its clinical presentation, progress, and outcome, and there is no consensus on its clinical management.

**Methods:**

To identify the characteristics of aHUS in Turkish children, an industry-independent registry was established for data collection that includes both retrospective and prospective patients.

**Results:**

In total, 146 patients (62 boys, 84 girls) were enrolled; 53 patients (36.3%) were less than 2 years old at initial presentation. Among the 42 patients (37.1%) whose mutation screening was complete for *CFH*, *CFI*, *MCP*, *CFB, C3*, *DGKE*, and *CHFR5* genes, underlying genetic abnormalities were uncovered in 34 patients (80.9%). Sixty-one patients (41.7%) had extrarenal involvement. During the acute stage, 33 patients (22.6%) received plasma therapy alone, among them 17 patients (51.5%) required dialysis, and 4 patients (12.1%) were still on dialysis at the time of discharge. In total, 103 patients (70.5%) received eculizumab therapy, 16 of whom (15.5%) received eculizumab as a first-line therapy. Plasma therapy was administered to 84.5% of the patients prior to eculizumab. In this group, renal replacement therapy was administered to 80 patients (77.7%) during the acute period. A total of 3 patients died during the acute stage. A total of 101 patients (77.7%) had a glomerular filtration rate >90 mL/min/1.73 m^2^ at the 2-year follow-up.

**Conclusions:**

The Turkish aHUS registry will increase our knowledge of patients with aHUS who have different genetic backgrounds and will enable evaluation of the different treatment options and outcomes.

## Background

Atypical hemolytic uremic syndrome (aHUS) is a life-threatening systemic disease associated with the dysregulation of the complement system [1]. It has been reported that mortality was higher in children when compared to adults whereas progression to end stage renal disease after the first episodes was higher in adults [2]. Mutations in the genes encoding complement regulatory proteins (i.e. *CFH*, *CFI*, *MCP (CD46)*, or *CFH-CFHR* genomic rearrangements) and components of the alternative pathway C3 convertase (i.e. *C3* and *CFB*) or anti-complement factor H autoantibodies are identified in 60–70% of patients with aHUS [2–7]. Recent studies have also identified mutations in diacylglycerol kinase-ε (*DGKE*), thrombomodulin (*THBD*, *CD141*) in thrombotic microangiopathies (TMAs) including aHUS [8–13]. For years, plasma therapy was the mainstay of treatment for aHUS [11, 12]. Eculizumab, a monoclonal antibody that blocks the terminal part of the complement system, has revolutionized the prognosis of aHUS and is currently recommended as first-line therapy in children with aHUS, whenever possible [11]. Several retrospective and prospective studies involving both pediatric and adult patients have demonstrated the efficacy and safety of eculizumab [11, 14, 15]. However, exact duration of therapy and dose intervals are still a matter of debate.

Owing to the low incidence of aHUS, large patient registries are required to adequately address such questions and to evaluate the natural history and progression of the disease, and the prognosis of patients from different ethnic backgrounds. One of the largest aHUS registries, the global aHUS registry, was established in 2012 with industry support; it includes multiple participating centers from 16 countries, not including Turkey. This registry prospectively collects information on patient demographics, disease characteristics, and treatment modalities [16]. In addition, there are a few registries of patients from different ethnic backgrounds [2, 17, 18]. However, investigators are aware that underlying genetic abnormalities of patients from different ethnic backgrounds differ and that the characteristics of aHUS may vary among different patient populations. In Turkey, an industry-independent aHUS registry was established to collect data on pediatric patients with aHUS who were treated in the 26 pediatric nephrology centers. This registry not only collects information on the demographic, clinical, laboratory, and genetic features of Turkish pediatric patients with aHUS, but also affords researchers a unique opportunity to evaluate the treatment strategies used at the different centers and to assess their impact on the long-term prognosis of the patients. This study aimed to introduce the registry and to provide a general perspective about its initial findings. Specific analyses addressing specific questions (i.e. genetics, long-term prognosis etc.) will be conducted in our subsequent studies.

## Methods

### Definitions

Pediatric aHUS was defined as a triad of Coombs-negative microangiopathic hemolytic anemia, thrombocytopenia, and acute renal failure in patients younger the age of 18 years. No adult patient, even if the first episode was observed during the childhood period, was included. Shiga toxin-producing *E. coli* (STEC) was investigated in a national centralized reference laboratory and STEC positive patients were excluded. Patients with depressed ADAMTS13 levels (i.e. ≤5%), other specific infections, co-existing diseases or those with drug-related HUS were also excluded [11].

Hematologic remission was defined as platelet count ≥150,000/mm^3^ and lactate dehydrogenase levels ≤ upper limits of normal for ≥2 consecutive measurements taken ≥4 weeks apart, and cessation of hemolysis [11].

Renal remission was defined as estimated glomerular filtration rate (eGFR) >90 mL/min/1.73 m^2^.

### Data collection

Beginning year 2013, all retrospective and prospective pediatric patients with aHUS from 26 pediatric nephrology centers have been registered in the database at www.ahusnet.org, a web-based national registry system (NRS). A pediatric nephrology specialist from each participating center is responsible for entering patients into the registry. The objectives of the NRS are to assess the clinical and genetic characteristics, treatment modalities, associated extrarenal findings, and clinical outcomes of patients with aHUS during their initial hospital admission, and to evaluate their long-term prognosis according to disease management strategies.

Responsible specialists from each center registered both retrospective and prospective patients in the secure NRS and were able to access the website at any time to enter and edit data. During patient registration, the following data were entered into the system: demographics, medical and disease history, clinical characteristics at disease onset (i.e. physical examination findings and laboratory data), genetic results (if available), all therapies implemented during the acute stage (i.e. plasma therapy including plasma infusions and/or plasma exchange, eculizumab, rituximab, corticosteroid, antihypertensive, hemodialysis, peritoneal dialysis, and continuous renal replacement therapies), and the renal/hematologic status (i.e. proteinuria, hypertension, serum creatinine level, hemoglobin level, and platelet count) of the patient at discharge. The side effects related to any of the treatment medications were also recorded in the NRS. Follow-up data, including the hematologic and renal parameters of the patient, current therapies, and relapses (if any, since the last visit), were entered into the NRS every 3 months. All patients in the NRS were evaluated annually by the joint committee, which comprised pediatric nephrology experts. Any patient who did not meet the criteria for aHUS, as determined by the joint committee, was removed from the registry.

### Genetic analyses and ELISA

Genetic testing was not a prerequisite for patient registration. However, if individual genetic data was available, their registration was also requested. DNA sample was available in 113 patients. Mutation analyses via Sanger sequencing of the coding regions of all of the following genes were performed in 42 patients (37.1%) at the Nephrogenetics Laboratory of Hacettepe University: *CFH*, *CFI*, *MCP*, *CFB, C3*, *DGKE*, and *CHFR5*. Where applicable, *in slico* analyses, using Sorting Tolerant From Intolerant (SIFT) (http://sift.jcvi.org), Polymorphism Phenotyping v2 (PolyPhen2) (http://genetics.bwh.harvard.edu/pph2/index.shtml), Mutation taster (http://www.mutationtaster.org), and Human Splicing Finder (http://www.umd.be/HSF3/index.html) softwares were applied for predicting likely effects of the variations. aHUS mutation database (http://www.fh-hus.org), The Human Gene Mutation Database Professional (http://www.hgmd.cf.ac.uk/ac/index.php) and dbSNP database (https://www.ncbi.nlm.nih.gov/snp) were used to check whether identified variations had been reported previously. *CFHR1-3* deletion in patients with anti-CFH autoantibodies was evaluated by multiplex ligation-dependent probe amplification (MLPA) analyses. Anti-complement Factor H autoantibody was searched in 44 patients using the CFH IgG ELISA Kit (Abnova™), according to the manufacturer’s recommendations, with a detection limit of 0.6 AU/mL. ADAMTS13 activity was detected using the ADAMTS-13 Activity Kit (Technozym™) according to the manufacturer’s recommendations, with a detection limit of 0.2% and an assay range of 0.3–105%.

The study was approved by the ethics committee of Hacettepe University (FON10/03-22). Written informed consent was obtained from the parents of all the patients.

### Statistical analysis

Descriptive statistical analysis methods were used to evaluate the demographics and clinical data. The mean, median, standard deviation, and interquartile range (IQR) were calculated for the numeric variables. Frequency tables were used to describe the categorical data. Mann Whitney *U* test was used to compare 2 independent samples. Data were analyzed using SPSS v.21 (SPSS Inc., Chicago, IL, USA).

## Results

### Patient characteristics

As of March 30, 2016, 146 patients (62 boys, 84 girls) from 26 pediatric nephrology centers were enrolled in the NRS. The demographic and clinical characteristics of the patients are presented in Table 1. A total of 69 patients (47.6%) had diarrhea prior to their initial presentation, and 35 patients (24.1%) had preceding upper respiratory tract infection. Notably, 53 of the 146 patients (36.3%) were aged less than 2 years at the first presentation, and 29 of the patients (19.8%) were aged less than 1 year. A total of 61 patients (41.7%) showed extra-renal involvement; the most common site of extra-renal manifestation was the neurologic system (*n* = 41; 28.1%). Hemiparesis, loss of vision, unconsciousness, headache, hallucination, encephalopathy were the main symptoms for the neurological involvement. In 8 of these patients, another system was also involved. Brain imaging was performed in 27 patients, 15 of whom showed abnormal radiological findings. Cardiac complications were identified in 9 patients (6.1%); 2 patients had hypertrophic cardiomyopathy, and the other cardiac manifestations included dilated cardiomyopathy, intracardiac thrombus, elevated creatine kinase (CK)-MB levels, mitral and tricuspid insufficiency, aortic valve insufficiency, left ventricular hypertrophy, and tachycardia. Involvement of the gastrointestinal system was observed in 16 children (10.9%) and pancreatitis, elevated liver enzymes, invagination, ischemic hepatitis, cholelithiasis, vomiting were the main symptoms. GI bleeding and pancreatitis were the most common findings, which were observed in 4 and 3 patients, respectively. Respiratory involvement was observed in 10 patients (6.8%), while 16 patients (10.9%) showed multisystem involvement. Kidney biopsies were performed in 44 patients. The biopsies were performed to confirm the diagnosis (in 29 patients), before initiation of eculizumab therapy (in 7 patients), to assess patients with nephrotic-range proteinuria (in 6 patients), and to evaluate patients who exhibited a poor response to plasma therapy or eculizumab (in 2 patients).

**Table 1:** Demographic and clinical characteristics of 146 patients with aHUS

Male (%) / Female (%)	62 (42.4) / 84 (57.6)
Current age, years	
Mean (SD)	7.98 (5.0)
Median (IQR)	6.53 (3.8–11)
Age at diagnosis, years	
Mean (SD)	4.82 (4.4)
Median (IQR)	3.5 (1.2–7.4)
Age category (years) at the time of diagnosis, n (%)	
<2	53 (36.3)
2 to <5	40 (27.4)
5 to <12	38 (26)
≥12	15 (10.3)
Duration of follow-up (years)	
Mean (SD)	2.9 (2.5)
Median (IQR)	2.1 (1.2–3.8)
Consanguinity (%)	42 (28.8)
Family history of aHUS (%)	7 (4.8)
Anuria (%) /oliguria at admission (%)	41 (28) / 67 (45.8)
Duration of anuria at admission (days)	
Mean (SD)	6.3 (4.5)
Median (IQR)	5 (2–10)
Duration of oliguria at admission (days)	
Mean (SD)	6 (6.8)
Median (IQR)	3 (2–7)
Extrarenal involvement, n (%)	61 (41.7)
Central nervous system	41 (28.1)
Gastrointestinal system	16 (10.9)
Cardiac	9 (6.1)
Respiratory system	10 (6.8)
Patients with GFR <90 mL/min/1.73 m^2^ at admission, n (%)	139 (95.2)
Hypocomplementemia (%)^a^	66 (48.5)
Renal biopsy at admission (%)	44 (30.1%)

SD: standard deviation; IQR: interquartile range; aHUS: atypical hemolytic uremic syndrome; GFR: glomerular filtration rate. ^a^Data is available for 136 patients

### Genetics

Number of mutation analyses performed in individual genes are as follows in 113 patients whose DNA sample was available: *CFH* (*n* = 64, 56.6%), *CF1* (*n* = 69, 61.0%), *MCP* (*n* = 49, 43.3%), *CFB* (*n* = 62, 54.8%), *C3* (*n* = 35, 30.9%), *DGKE* (*n* = 67, 59.2%). Mutation screening was complete for all of the corresponding genes in 42 patients (37.1%), and underlying genetic abnormalities were uncovered in 34 of these patients (80.9%). These include isolated abnormality in *MCP* (*n* = 7, 16.3%), in *DGKE* (*n* = 6, 8.9%), in *C3* (*n* = 4, 11.4%), in *CFH* (*n* = 5, 7.8%), in *CFB* (*n* = 1, 1.6%), and in *CFI* (*n* = 1, 1.4%). In 4 patients, combined genetic abnormalities were detected: *CFH/CFB* mutation (*n* = 1), *CFB* mutation/*CFHR1-3* deletion (n =1), *CFB* mutation/*CFHR1-3* deletion/anti-CFH autoantibody (*n* = 1), and *CFHR5* mutation/*CFHR1-3* deletion/anti-CFH autoantibody (*n* = 1). In addition, 1 patient was diagnosed with cobalamin deficiency. The anti-CFH autoantibody was detected in 5 out of 44 patients who had been studied for anti-CFH autoantibody (11.3%). All of these 5 patients had homozygous *CFHR1-3* deletion. To our best of knowledge, 17 novel variations have been identified in this study (Table 2).

**Table 2:** Genetic results of the patients

Gene	Screened n (%)^a^	Variation n (%)^b^	Variant (dbSNP database)	Predicted aminoacid change	Zygosity	MAF_1000G	MAF_ESP 6500	SIFT prediction^c^ (score)	PolyPhen prediction^d^ (score)	Mutation Taster prediction^e^ (score)	HSF prediction^f^	Ref
*CFH (NM_000186.3)*												
	64 (56.6)	5 (7.8)										
			c.3530A>G	p.Tyr1177Cys	Hom	NR	NR	Tolarated (0.19)	Probably damaging (0.996)	Polymorphism (0.99)	NA	[22]
			c.2850G>T (rs149474608)	p.Gln950His	Het	<0.01	<0.01	Deleterious (0.049)	Probably damaging (0.96)	Polymorphism (0.99)	NA	[26]
			c.2127_2129del	p.Tyr711del	Het	NR	NR	NA	NA	Disease causing (0.54)	NA	Novel
			c.3148A>T (rs35274867)	p.Asn1050Tyr	Het	0.01	0.01	Tolarated (0.08)	Benign (0.016)	Polymorphism (0.99)	NA	[27]
			c.3133+1G>A	Splice site	Het	NR	NR	NA	NA	N/A	Broken WT Donor Site	Novel
*CFI (NM_000204.3)*												
	69 (61.0)	1 (1.4)										
			c.608C>T (rs138346388)	p.Thr203Ile	Het	<0.01	<0.01	Tolarated (0.16)	Benign (0.051)	Polymorphism (0.99)	NA	[28]
*MCP (NM_002389)*												
	49 (43.3)	7 (14.2)										
			c.535G>C (rs779174212)	p.Glu179Gln	Hom	<0.01	<0.01	Tolarated (0.33)	Benign (0.023)	Polymorphism (0.99)	NA	[29]
			c.841C>T	p.Pro281Ser	Hom	NR	NR	Damaging (0)	Probably damaging (0.99)	Disease causing (0.85)	NA	Novel
			c.841C>T	p.Pro281Ser	Het	NR	NR	Damaging (0)	Probably damaging (0.99)	Disease causing (0.85)	NA	Novel
			c.476-2A>G	Splice site	Hom	NR	NR	NA	NA	NA	Broken WT Acceptor Site	Novel
			c.476-2A>G	Splice site	Hom	NR	NR	NA	NA	NA	Broken WT Acceptor Site	Novel
			c.286+2T>G	Splice site	Hom	NR	NR	NA	NA	NA	Broken WT Donor Site	[30]
			c.1027+5G>T	Splice site	Hom	NR	NR	NA	NA	NA	Broken WT Donor Site	[31]
*CFB (NM_001710)*												
	62 (54.8)	1 (1.6)										
			c.125T>C	p.Val42Ala	Het	NR	NR	Tolarated (0.19)	Benign (0.12)	Disease causing (0.94)	NA	Novel
*C3 (NM_000064)*												
	35 (30.9)	4 (11.4)										
			c.537_539del	p.Leu180del	Het	NR	NR	NA	NA	Polymorphism (0.99)	NA	Novel
			c.3125G>T	p.Arg1042Leu	Het	NR	NR	Damaging (0.03)	Probably damaging (0.99)	Disease causing (0.99)	NA	[17]
			c.4148C>A (rs139100972)	p.Thr1383Asn	Het	NR	<0.01	Tolarated (0.5)	Benign (0.36)	Polymorphism (0.99)	NA	[3]
			c.1976-6C>T	Splice site	Het	NR	NR	NA	NA	NA	Probably no impact	Novel
*DGKE(NM_003647)*												
	67 (59.2)	6 (8.9)										
			c. 1009C>T (rs762576212)	p.Arg337Stop	Hom	NR	<0.01	NA	NA	Disease causing (1)	NA	in dbSNP
			c.118_121dup	p.Ser41Metfs*2	Hom	NR	NR	NA	NA	Disease causing (1)	NA	Novel
			c.607_610del	p.Lys203Glnfs*6	Hom	NR	NR	NA	NA	Disease causing (1)	NA	Novel
			c.263_264insGGG	p.Asp88Glufs*84	C-Het	NR	NR	NA	NA	Disease causing	NA	Novel
			CGCCA/c.76del	p.Thr26Argfs*143		NR	NR	NA	NA	Disease causing (1)		Novel
			c. 427C>T	p.Gln143Stop	C-Het	NR	NR	NA	NA	Disease causing	NA	Novel
			c.1133C>G	p.Pro378Arg		NR	<0.01	Damaging (0)	Possibly damaging (0.93)	Disease causing (1)		Novel
			c.793A>C	p.Thr265Pro	Het	NR	NR	Tolarated (0.08)	Benign (0.43)	Disease causing (0.99)	NA	Novel
*Combined genetic abnormalities*												
*CFH*			c.3644G>A (rs121913051)	p.Arg1215Gln	Het	NR	NR	Tolarated (0.19)	Probably damaging (0.99)	Polymorphism (0.99)	NA	[32]
CFB			c.1135C>T	p.Arg379Cys	Het	NR	NR	Damaging (0.01)	Probably damaging (0.99)	Polymorphism (0.97)	NA	Novel
CFB			c.1050G>T	p.Lys350Asn	C-Het	NR	NR	T olarated (0.14)	Probably damaging (0.99)	Polymorphism (0.81)	NA	[33]
			c.1697A>C (rs45484591)	p.Glu566Ala		0.01	0 . 0 1	Tolarated (0.73)	Benign (0)	Polymorphism (0.99)	NA	[34]
Heterozygous CFHR1-3 deletion												
CFB			c.397G>A	p.Asp133Asn	Het	NR	NR	Damaging (0.05)	Benign (0.014)	Polymorphism (0.7)	NA	Novel
Anti-CFH antibody associated with homozygous CFHR1-3 deletion												
*CFHR5 (NM_030787)*			c.53dup	p.Glu19Argfs*6	Het	NR	NR	NA	NA	Disease causing (1)	NA	Novel
Anti-CFH antibody associated with homozygous CFHR1-3 deletion												

^a^of the 113 patients whose DNA samples were avaliable, ^b^of the analyses that were performed

^c^Sorting Tolerant From Intolerant (SIFT) (http://sift.jcvi.org); ^d^Polymorphism Phenotyping v2 (http://genetics.bwh.harvard.edu/pph2/index.shtml);

^e^Mutation taster (http://www.mutationtaster.org);^f^Human Splicing Finder (http://www.umd.be/HSF3/index.html)

Variations and predicted aminoacid changes have been named according to the guidelines of the Human Genome Variation Society using Mutalyzer software (https://mutalyzer.nl)

C-Het, compound heterozygous; Het, heterozygous; Hom, homozygous; NA, not applicable; NR, not reported; WT, wild-type

MAF-1000G, minor allele frequency based on 1000 Genomes Project

MAF-6500, minor allele frequency based on 6503 samples collected at NHLBI Exome Sequencing Project and data from 60.706 individuals aggregated by the Exome Aggregation

Consortium (ExAC; http://exac.broadinstitute.org); (accessed October 2016)

### Treatment

Remission was achieved with supportive therapy in 5 patients and by cobalamin administration, in 1 patient. Four patients were diagnosed with ESKD at the time of admission and, therefore, did not receive specific therapy. During the acute stage, 33 patients (22.6%; M/F 13/20) were treated with plasma therapy alone (i.e. plasma infusion and/or plasma exchange). Of these, 15 patients received plasma infusions, 14 patients underwent plasma exchanges, and 4 patients received both therapies. The median age at diagnosis in this group of patients was 3.9 (IQR 1.8–8.3) years. In total, 17 patients (51.5%) required dialysis; 2 patients (6%) died during the acute stage. At the time of discharge, 4 patients (12.1%) were on dialysis, 26 patients (78.7%) did not require dialysis, and data on 1 patient were not available. The median follow-up time for this group of patients was 1.7 (IQR 1.1–5.3) years. None of the patients required plasma therapy after discharge. At the last follow-up, 1 patient was still on dialysis and 1 patient had undergone renal transplantation; no other renal replacement therapies were recorded for the remaining patients. At the last follow-up visit, the GFR of 23 of the patients (69.6%) was >90 mL/min/1.73 m^2^.

In this registry population, 103 patients (70.5%) received eculizumab therapy (45 males, 58 females). The median age at diagnosis in this group of patients was 3.4 (IQR 1.2–7) years, and the median follow-up duration was 2.05 (IQR 1.1–3.2) years. Eighty patients (77.7%) underwent renal replacement therapy during the acute period. Plasma therapy was administered to 84.5% of the patients prior to the initiation of eculizumab, and 16 patients (15.5%) received eculizumab as a first-line therapy. Complete hematologic and renal remission was achieved in 82.2% and 76.3% of the patients, respectively. Median follow up duration was 2.05 years (IQR 1.1-3.2 years) for this group. During the follow-up period, eculizumab treatment was discontinued in 52 patients (51.5%) for various reasons (drug unavailability, genetic background of the patient, choice of the patient’s family, or the clinician’s decision). Five patients (4.8%) with severe renal disease did not respond to therapy and progressed to ESKD. The initial median eGFR of these patients was significantly low when compared with the eGFRs of the rest of the patients (7.6 mL/min/1.73 m^2^ [range, 5.9–18.3 mL/min/1.73 m^2^] vs. 18.6 mL/min/1.73 m^2^ [range, 0.3–166 mL/min/1.73 m^2^]; *P* = 0.019). At the last follow-up visit, the GFR of 68 of the patients (66%) was >90 mL/min/1.73 m^2^. Eculizumab-related side effects were reported in 2 patients: an allergic reaction in 1 patient, and convulsions and sepsis after eculizumab administration in 1 patient. No patient relapsed while receiving eculizumab therapy.

### Follow-up

A total of 3 patients died during the acute stage. The causes of death included sepsis (*n* = 1), intractable seizures (*n* = 1), and multisystem involvement (*n* = 1). Three patients died during the follow-up period. A total of 10 patients were lost to follow-up, and outcome data were available for the remaining 130 patients (56 males, 74 females). At their last follow-up visit, 13 patients had undergone treatment for ESKD (hemodialysis [*n* = 5], peritoneal dialysis [*n* = 3], and renal transplantation [*n* = 5]). More than 75% of the patients had a GFR >90 mL/min/1.73 m^2^ (Table 3).

**Table 3:** Follow-up characteristics of patients (*n* = 130)

Age at diagnosis, years	
Mean (SD)	4.8 (4.4)
Median (IQR)	3.5 (1.2–7.1)
Duration of follow-up (years)	
Mean (SD)	2.8 (2.3)
Median (IQR)	2.1 (1.2–3.7)
Renal replacement therapy^a^, n (%)	13 (10)
GFR (mL/min/1.73 m^2^) at the last visit, n (%)	
>90	101 (77.7)
60–89	14 (10.8)
30–59	6 (4.6)
15–29	3 (2.3)
0–14	6 (4.6)

*SD* standard deviation, *IQR* interquartile range, *GFR* glomerular filtration rate

^a^Includes hemodialysis, peritoneal dialysis, and renal transplantation

## Discussion

Here, we introduce one of the largest pediatric aHUS registries in the world. However, it is very well known that registries have its own limitations. In aHUS, many factors such as STEC, genetics, anti-factor H antibodies and follow-up data of the patients have to be taken into consideration to draw a certain conclusion. In this study, we performed longitudinal follow-ups of pediatric patients with aHUS to evaluate their clinical and genetic characteristics in order to better understand the diversity of this rare disease in the Turkish population. Owing to the low incidence of aHUS, majority of the previous series on aHUS have included both adult and pediatric patients [2, 16–18] However, pediatric aHUS cases have different disease characteristics than those of adult cases. A study by Fremeaux-Bacchi et al. consisted of 214 patients, 89 of whom were pediatric patients. In this study, the characteristics of adult and pediatric patients were compared [2]. Another analysis of aHUS was from the global aHUS registry, which was supported by an industry sponsor. In that study, the baseline demographic and clinical characteristics of 516 patients with aHUS from 195 clinical centers in 16 countries were described. In that series, 201 patients were in the pediatric age group [16]. When compared with other established series, the Turkish aHUS registry has several distinctive features, namely its homogenous nature (i.e. it involves only Turkish patients with aHUS); availability of detailed data on patient demographics, initial clinical presentation, and the different treatments employed at the clinical centers; and availability of long-term follow-up and outcome data.

In our study, there were more female patients than male patients. While the mean patient age at diagnosis was comparable to the mean age of the population of the global aHUS registry, it was higher than the mean age of the population in the French registry (4.8 years vs. 1.5 years). Patients aged younger than 2 years accounted for 36.3% of the population in our study, which was significantly lower than the corresponding percentages reported in the global and French registries (43.9% and 56%, respectively) [2, 16]. A total of 28 patients (19.1%) in our study were aged under 1 year; the corresponding data are not available in the other registries. In our study, patients aged older than 12 years constituted 10.3% of all patients, whereas this figure was 6.5% in the global registry. All these discrepancies could be attributed to the different genetic abnormalities and/or environmental factors that the patients in the different populations were exposed to. It is well known that some genetic abnormalities are associated with early-onset aHUS whereas others can cause adolescent- or even adult-onset aHUS. One of the most striking findings in our study was the rather low percentage of patients with a family history of aHUS when compared to the global registry (4.8% vs 20.4%) despite a high rate of consanguinity in the Turkish population [16]. This was an unexpected finding and may be due to the fact that family history was not examined thoroughly by the reporting clinicians in our study. Alternatively, since the diagnosis of aHUS requires specific knowledge and expertise, aHUS among family members of the Turkish registry patients may not have been recognized if they did not undergo a specialist evaluation.

Extra-renal involvement in aHUS can significantly impact patient prognosis. Neurologic involvement is the most frequent complication, which can occur in 8–30% of the patients [10]. In our study, neurologic involvement was observed in 28.1% of the patients, which is consistent with the literature. The involvement of other organ systems is reported to be much lower than that of the central nervous system. Johnson et al. reported that the most common gastrointestinal manifestations were pancreatitis/pancreatic insufficiency and elevated transaminases, and they observed respiratory and cardiac involvement (i.e. heart failure/cardiomyopathy and pericardial effusion) in 21% and 5.6% of pediatric patients with aHUS, respectively [12]. In the current study, we observed that pancreatitis was one of the most common gastrointestinal system complications and that cardiac involvement occurred at a similar rate (6.1%), whereas respiratory involvement was slightly lower (6.8%).

Although we have limited data on genetics, the results of our study might provide a general idea of the underlying genetic abnormalities in Turkish patients with aHUS. It has recently been reported that 60–70% of patients with aHUS carry identifiable mutations in complement genes or anti-CFH antibodies, which result in TMA lesions [11]. In a multicenter audit analysis comprising 71 patients, 61% of the patients with results available for both complement genetic testing and anti CFH-autoantibody testing had evidence of alternative complement pathway dysregulation [12]. In our study population, genetic analyses were completed for the corresponding genes in 42 patients (37.1%). In this subset of patients, disease-causing mutations, 17 of which were novel, were identified in 34 patients (80.9%). This figure might indicate that the probability of detecting any mutation in already known genes is high in Turkish patients and, thereby, justifies the necessity of genetic screening for a long-term therapeutic plan. In previous studies, *CFH* mutations were the most commonly identified abnormalities [2, 18]. In contrast, we found that *MCP* mutations and *C3* mutations were the most common genetic abnormalities whereas *CFH* mutations were detected in less than 10% of the genetically studied population. These initial results may confirm the racial differences between Turks and other populations. One of the limitations of our study was that we did not screen for thrombomodulin mutations, which have a minor role in aHUS [11].

Before the introduction of eculizumab, plasma therapy was the mainstay of treatment for aHUS, based on the premise that it was beneficial by supplying normal complement proteins and/or removing mutant proteins or autoantibodies [11, 12]. After the study published by Bell et al. in 1991, plasma-based therapies became the initial treatment for most patients with aHUS [19]. Noris et al. reported that two-third of adult patients with aHUS had a poor outcome after 3 years of follow-up [3]. In a study by Johnson et al. on aHUS treatment outcomes in 71 patients, 59 patients received plasma therapy and the other 12 patients received other types of treatment. Their results revealed that 17% of the patients remained dependent on dialysis and 80.3% of the patients had persistent renal sequelae at day 33 of treatment. The long-term results of plasma therapy were unavailable [12]. In our study, 12.1% of the patients were on dialysis at the time of discharge; after a median follow-up period of 1.7 years, 1 patient was still on dialysis and 1 patient had undergone renal transplantation. These differences in findings between our study and other studies may be attributed to the different genetic backgrounds of the studied populations. Plasma therapy (i.e. plasma infusions and/or plasma exchange) still appears to be a valid therapeutic option for Turkish patients with aHUS.

The introduction of eculizumab has profoundly changed the management of aHUS. The efficacy of eculizumab has been demonstrated in three prospective clinical trials performed primarily in adult patients with TMA [14, 20, 21]. In these trials, it was demonstrated that eculizumab resulted in a time-dependent improvement in patients with aHUS, and the beneficial effects were maintained after 2 years of follow-up. In children, the evidence for the efficacy of eculizumab came from case reports and retrospective studies [11, 22, 23]. Recently, Greenbaum et al. published the first pediatric prospective study that assessed the efficacy and safety of eculizumab in patients with aHUS who were aged <18 years. Mutations in the genes of the alternative complement pathway were identified in 50% of the patients [15]. *MCP* mutations were the most common; the mean duration of eculizumab treatment was 5.5 months, and hematological remission occurred in 82% of the patients. The authors reported that the mean improvement in eGFR from baseline to week 27 was 64 mL/min/1.73 m^2^, and discontinuation of dialysis was possible in 82% of the patients who initially required renal replacement therapy [15]. In our study, 16 patients (15.5%) received eculizumab as a first-line therapy. Complete hematologic and renal remission was achieved in 82.2% and 76.3% of the patients, respectively. Our study as well as the study by Greenbaum et al. supported the efficacy of eculizumab in the acute period. However, there is ongoing debate on the appropriate duration and intervals for eculizumab treatment. Ardissino et al. reported findings from 10 patients who received eculizumab treatment, which was discontinued later. All of these patients had complement system dysregulation, and showed beneficial effects after being treated with eculizumab. After the eculizumab treatment was stopped, 3 patients relapsed within 6 weeks [24]. Later, the authors published results from a longer follow-up period. During a cumulative time off treatment of 243 months, 5 of the patients experienced relapse within 6 months of their last eculizumab dose [25]. In our patient cohort, eculizumab was discontinued in 52 patients for various reasons. The analyses of the genetic backgrounds and clinical outcomes of the patients are still underway and will be reported in detail in another manuscript.

Another noteworthy feature of our study is that we completed almost 2 years of patient follow-up. It has been previously reported that the mortality rate in children with aHUS was higher than that in adults, but the renal outcomes were worse in adults than in children [2]. In our study on pediatric patients, the mortality rate was 2% (3 out of 146 patients) during the acute stage, and 77.7% of the patients had a GFR >90 mL/min/1.73 m^2^ during the follow-up period. Our results suggest that aHUS is a treatable disease and that progression to ESKD can be prevented with early diagnosis, and meticulous treatment and follow-up. However, it should be noted that in our patient cohort, high frequency of *MCP* mutations, lack of information on completed genetic data and early initiation of treatment may explain favorable outcome.

## Conclusions

We have established a unique Turkish aHUS registry that will increase our knowledge of patients with aHUS who have different genetic backgrounds, and enable the evaluation of different treatment options and patient outcomes. This registry is now open to adult patients. The collection of data on adult patients with aHUS will help researchers to better characterize the spectrum of adult aHUS in the country and will enable comparisons with pediatric cases.

## Abbreviations

aHUS: Atypical hemolytic uremic syndrome
C3: Complement factor 3
CFB: Complement factor B
CFH: Complement factor H
CFI: Complement factor I
CK: Creatine kinase
CFH-CFHR: Complement factor H-complement factor H related proteins
DGKE: Diacylglycerol kinase-ε
eGFR: Estimated glomerular filtration rate
ESKD: End-stage kidney disease
GI: Gastrointestinal
IQR: Interquartile range
MCP (CD46): Membrane cofactor protein
MLPA: Multiplex ligation-dependent probe amplification
NRS: National registry system
SD: Standard deviation
STEC: Shiga toxin-producing *E. coli*
THBD: Thrombomodulin
TMA: Thrombotic microangiopathy
